# Two Models, One Waiting List: A Service Evaluation Comparing In-House and Outsourced Management

**DOI:** 10.1192/bjo.2026.11588

**Published:** 2026-06-30

**Authors:** Christina Sim

**Affiliations:** HSE, Galway, Ireland

## Abstract

**Aims:**

Prolonged waiting times for ADHD assessment in child and adolescent mental health services (CAMHS) present a significant challenge. While outsourcing can address immediate waiting list pressures, it may simply shift the bottleneck back to the core CAMHS service. This service evaluation aimed to examine the effectiveness and cost implications of the in-house waiting list model in West Galway CAMHS, with a view to informing management decisions on developing and appropriately resourcing the team for sustainable long-term service delivery.

**Methods:**

This service evaluation reviewed CAMHS patients from West Galway who were awaiting ADHD assessment between January 2025 and January 2026. A random sample of 30 patients managed via the in-house waiting list initiative was compared with 28 patients managed through an outsourced model. Outcomes assessed included time to assessment, discharge to GP (without transfer to core CAMHS), diagnosis rate, and subsequent need for CAMHS follow-up. Data were analysed using simple descriptive comparisons to explore differences in service outcomes between the two models.

**Results:**

Direct comparison of time to assessment was limited by differences in team structure and staffing: the in-house waiting list team was newly established and consultant-led, while the outsourced model comprised a well-established external agency with longstanding experience in the UK. Notably, 70% of patients in the in-house team were discharged back to their GP without transfer to core CAMHS, compared with 0% in the outsourced group. Consequently, only 30% of in-house patients required ongoing CAMHS follow-up, versus 100% in the outsourced model. Diagnosis rates are currently being further analysed to examine the detection of comorbidities. These preliminary findings suggest that while outsourcing addresses immediate assessment capacity, the in-house model may offer opportunities for developing a sustainable, consultant-led service with longer-term oversight, provided adequate resourcing.

**Conclusion:**

This service evaluation highlights that a well-structured in-house waiting list team has the potential to improve downstream flow to the core CAMHS service, reducing the secondary bottleneck and minimising repetition of work through clear, consultant-led care plans. Effectiveness is dependent on having the right staffing skill set and staff-to-patient ratio, as limitations in these areas impact throughput, consistency, and the ability to meet service expectations across teams. These findings suggest that appropriately resourced, consultant-led in-house services could provide sustainable improvements in assessment and care delivery, supporting the case for expanding the service.

